# Supplementary material for: Resolving the full spectrum of human genome variation using Linked-Reads
Source: Genome Res. 2019 Apr;29(4):635–45. doi: 10.1101/gr.234443.118 (PMC6442396; doi:10.1101/gr.234443.118)
Supplement: Supplemental Material [file supp_gr.234443.118_Supplemental_longranger-2.2.zip › longranger-2.2.tar.bz2!/lr2.2-public/lib/stan/cmdstan/stan/doc/api/html/structstan_1_1gm_1_1returns__type__vis.html]

Stan: stan::gm::returns\_type\_vis Struct Reference


|  |  |
| --- | --- |
|  | Stan  2.5.0  probability, sampling & optimization |


- Main Page
- Related Pages
- Namespaces
- Classes
- Files

- Class List
- Class Index
- Class Hierarchy
- Class Members

All Classes Namespaces Files Functions Variables Typedefs Enumerations Enumerator Friends Macros Pages

- stan
- gm
- returns\_type\_vis

Public Member Functions |
Public Attributes |
List of all members

stan::gm::returns\_type\_vis Struct Reference

`#include <ast.hpp>`

Inheritance diagram for stan::gm::returns\_type\_vis:

|  |  |
| --- | --- |
| Public Member Functions | |
|  | returns\_type\_vis (const expr\_type &return\_type, std::ostream &error\_msgs) |
|  | |
| bool | operator() (const nil &st) const |
|  | |
| bool | operator() (const assignment &st) const |
|  | |
| bool | operator() (const sample &st) const |
|  | |
| bool | operator() (const increment\_log\_prob\_statement &t) const |
|  | |
| bool | operator() (const expression &st) const |
|  | |
| bool | operator() (const statements &st) const |
|  | |
| bool | operator() (const for\_statement &st) const |
|  | |
| bool | operator() (const conditional\_statement &st) const |
|  | |
| bool | operator() (const while\_statement &st) const |
|  | |
| bool | operator() (const print\_statement &st) const |
|  | |
| bool | operator() (const reject\_statement &st) const |
|  | |
| bool | operator() (const no\_op\_statement &st) const |
|  | |
| bool | operator() (const return\_statement &st) const |
|  | |

|  |  |
| --- | --- |
| Public Attributes | |
| expr\_type | return\_type\_ |
|  | |
| std::ostream & | error\_msgs\_ |
|  | |

## Detailed Description

Definition at line 688 of file ast.hpp.

## Constructor & Destructor Documentation

|  |  |  |  |
| --- | --- | --- | --- |
| stan::gm::returns\_type\_vis::returns\_type\_vis | ( | const expr\_type & | *return\_type*, |
|  |  | std::ostream & | *error\_msgs* |
|  | ) |  |  |

Definition at line 428 of file ast\_def.cpp.

## Member Function Documentation

|  |  |  |  |  |  |
| --- | --- | --- | --- | --- | --- |
| bool stan::gm::returns\_type\_vis::operator() | ( | const nil & | *st* | ) | const |

Definition at line 433 of file ast\_def.cpp.

|  |  |  |  |  |  |
| --- | --- | --- | --- | --- | --- |
| bool stan::gm::returns\_type\_vis::operator() | ( | const assignment & | *st* | ) | const |

Definition at line 438 of file ast\_def.cpp.

|  |  |  |  |  |  |
| --- | --- | --- | --- | --- | --- |
| bool stan::gm::returns\_type\_vis::operator() | ( | const sample & | *st* | ) | const |

Definition at line 443 of file ast\_def.cpp.

|  |  |  |  |  |  |
| --- | --- | --- | --- | --- | --- |
| bool stan::gm::returns\_type\_vis::operator() | ( | const increment\_log\_prob\_statement & | *t* | ) | const |

Definition at line 448 of file ast\_def.cpp.

|  |  |  |  |  |  |
| --- | --- | --- | --- | --- | --- |
| bool stan::gm::returns\_type\_vis::operator() | ( | const expression & | *st* | ) | const |

Definition at line 454 of file ast\_def.cpp.

|  |  |  |  |  |  |
| --- | --- | --- | --- | --- | --- |
| bool stan::gm::returns\_type\_vis::operator() | ( | const statements & | *st* | ) | const |

Definition at line 475 of file ast\_def.cpp.

|  |  |  |  |  |  |
| --- | --- | --- | --- | --- | --- |
| bool stan::gm::returns\_type\_vis::operator() | ( | const for\_statement & | *st* | ) | const |

Definition at line 485 of file ast\_def.cpp.

|  |  |  |  |  |  |
| --- | --- | --- | --- | --- | --- |
| bool stan::gm::returns\_type\_vis::operator() | ( | const conditional\_statement & | *st* | ) | const |

Definition at line 493 of file ast\_def.cpp.

|  |  |  |  |  |  |
| --- | --- | --- | --- | --- | --- |
| bool stan::gm::returns\_type\_vis::operator() | ( | const while\_statement & | *st* | ) | const |

Definition at line 489 of file ast\_def.cpp.

|  |  |  |  |  |  |
| --- | --- | --- | --- | --- | --- |
| bool stan::gm::returns\_type\_vis::operator() | ( | const print\_statement & | *st* | ) | const |

Definition at line 459 of file ast\_def.cpp.

|  |  |  |  |  |  |
| --- | --- | --- | --- | --- | --- |
| bool stan::gm::returns\_type\_vis::operator() | ( | const reject\_statement & | *st* | ) | const |

Definition at line 464 of file ast\_def.cpp.

|  |  |  |  |  |  |
| --- | --- | --- | --- | --- | --- |
| bool stan::gm::returns\_type\_vis::operator() | ( | const no\_op\_statement & | *st* | ) | const |

Definition at line 469 of file ast\_def.cpp.

|  |  |  |  |  |  |
| --- | --- | --- | --- | --- | --- |
| bool stan::gm::returns\_type\_vis::operator() | ( | const return\_statement & | *st* | ) | const |

Definition at line 507 of file ast\_def.cpp.

## Member Data Documentation

std::ostream& stan::gm::returns\_type\_vis::error\_msgs\_

Definition at line 690 of file ast.hpp.

expr\_type stan::gm::returns\_type\_vis::return\_type\_

Definition at line 689 of file ast.hpp.

---

The documentation for this struct was generated from the following files:

- src/stan/gm/ast.hpp
- src/stan/gm/ast\_def.cpp


---

[ Stan Home Page ]

*© 2011–2014,
Stan Development Team.*
